# Family influences on oral PrEP use among adolescent girls and young women in Kenya and South Africa

**DOI:** 10.1371/journal.pone.0292529

**Published:** 2023-11-14

**Authors:** Makhosazane Nomhle Ndimande-Khoza, Ariana W. K. Katz, Sinead Moretlwe-Delany, Danielle Travill, Elzette Rousseau, Victor Omollo, Jennifer Morton, Rachel Johnson, Linda-Gail Bekker, Elizabeth A. Bukusi, Jared Baeten, Connie Celum, Ariane van der Straten, Sarah T. Roberts

**Affiliations:** 1 Wits RHI, Faculty of Health Sciences, University of the Witwatersrand, Johannesburg, South Africa; 2 RTI International, Women’s Global Health Imperative (WGHI), Berkeley, California, United States of America; 3 Desmond Tutu Health Foundation, University of Cape Town, Cape Town, South Africa; 4 Centre for Microbiology Research, Kenya Medical Research Institute, Nairobi, Kenya; 5 Departments of Global Health, Medicine, and Epidemiology, University of Washington, Seattle, WA, United States of America; 6 Gilead Sciences, Foster City, CA, United States of America; 7 Center for AIDS Prevention Studies, Department of Medicine, University of California, San Francisco, San Francisco, California, United States of America; 8 ASTRA Consulting, Kensington, CA, United States of America; Charite Universitatsmedizin Berlin, GERMANY

## Abstract

**Introduction:**

Effective use of oral HIV pre-exposure prophylaxis (PrEP) has been lower among African adolescent girls and young women (AGYW) than among older women, young men who have sex with men, and serodiscordant heterosexual couples in the region. Efforts to build PrEP support have centered around peers and male partners, but the family may also play an important role. This qualitative study aimed to describe family influence on PrEP use among AGYW in in three African cities.

**Methods:**

POWER (Prevention Options for Women Evaluation Research) was a PrEP demonstration project among 2550 AGYW (16–25 years old) in Johannesburg and Cape Town, South Africa and Kisumu, Kenya conducted from 2017 to 2020. In-depth interviews and focus group discussions were conducted with 136 AGYW participants to explore their PrEP views and experiences, including awareness and interest in PrEP; barriers and facilitators to uptake and use; the influence of family, peers, intimate partners, and community; and the key types of support for their PrEP use. Transcripts were coded and analysed thematically.

**Results:**

The decision to initiate PrEP was associated with fear and anxiety linked to anticipated stigma from family members, and with family’s lived HIV experience. Family disclosure, especially to mothers, was important to participants, as most lived with their families and considered it essential for them to obtain their mother’s approval to use PrEP. Most family members, particularly mothers, provided instrumental, emotional, informational and appraisal support to participants using PrEP, including reminders, encouragement, and problem-solving. Participants reported that family members with insufficient information about PrEP safety and efficacy and who voiced concerns were a substantial barrier to their use. However, they often became supportive after receiving more PrEP information.

**Conclusion:**

Families, particularly mothers, can play an important role in supporting PrEP use. PrEP programmes should leverage family support to help with PrEP persistence by providing basic information to families about PrEP safety and efficacy. AGYW using PrEP should be encouraged to selectively disclose PrEP use to build support and counseled on how to disclose and address family concerns.

## Introduction

In 2015, adolescent girls and young women (AGYW) in Eastern and Southern Africa accounted for 25% of all new HIV infections in this region and remain a priority for HIV prevention [1]. Oral pre-exposure prophylaxis (PrEP) holds promise for HIV prevention in this population, and demonstration projects conducted from 2016 to 2019 in South Africa, Kenya and Zimbabwe have shown high PrEP uptake, with 93–95% of AGYW accepting PrEP the first time it is offered to them [2, 3]. Data show only one-third of AGYW persist with PrEP use beyond three months, with as few as 6%-8% still using PrEP beyond 6 months after initiation [2, 4–6]. This suggest that AGYW are interested in PrEP and see the need for it but face challenges in using it.

Several individual and social factors contribute to low PrEP persistence. Individual factors include forgetfulness, pill fatigue, and logistical challenges such as getting to the clinic [7]. Anticipated and experienced stigma leads to covert PrEP use, which is associated with poor adherence [8]. Lack of disclosure and lack of social support from peers, partners, family, and the community are barriers to persistence, which may be compounded by the fear of or actual and violence towards those who disclose use [9–11]. Direct opposition (where family, partners, or other key influencers express negative comments or discourage or forbid PrEP use) following disclosure can also lead to PrEP discontinuation [12–14].

Social support, defined as the assistance individuals receive or perceive from their social networks [15, 16], is key to HIV treatment and prevention. Social support includes emotional support (care, empathy, trust and love), appraisal support (feedback, assessment), informational support (suggestion, advice, information), and instrumental support (tangible or practical aid and services such as money, time, and reminders) [17, 18]. A large body of evidence has identified social support networks as crucial influences on antiretroviral therapy (ART) adherence [19–21]. Similarly, social support has been shown to improve the impact of HIV prevention interventions [22–25]. Social support networks indirectly mitigate negative stressors such as fear and stigma, and increase self-efficacy [26, 27]. Disclosure is one way of eliciting social support [9, 19, 28]; however, it could also pose a risk of potential backlash such as stigma, intimate partner violence and other negative outcomes [29]. In a study conducted in South Africa, healthcare providers reported that young women living with HIV who disclosed ART use to their significant others and received social support were likely to have better ART adherence outcomes than those who did not disclose or disclosed but did not receive support [30]. These findings suggest receipt of social support has implications on adherence to medication.

Currently, there is limited research exploring the role of social influences on PrEP use among AGYW in eastern and southern Africa. Efforts to build social support for PrEP have centered around peers and male partners [31, 32], but family members may also play an important role, including, parents, siblings, other relatives and caregivers (aunts and grandmothers) who may or may not reside with the PrEP user [33]. Recent findings from 3P (perception, partners, pills), an HIV prevention study conducted among South African AGYW aged 16–25 showed that disclosure to family was associated with approximately six times higher odds of PrEP adherence [8]. These findings support the need to strengthen family relationships and promote social support within families of adolescents to address PrEP adherence challenges among adolescents in sub-Saharan Africa [34]. There is a need for similar support strategies that address AGYW’s PrEP adherence challenges. The few studies [9, 31] that have assessed the effects of disclosure on PrEP use, have not described the types of support AGYW received from their families. Research is needed to inform best practices for identifying support networks and leveraging support from PrEP allies to promote the formation and continuation of early adherence behaviors. Since most adolescents still live in their family homes, parents can strongly influence adolescent motivations, decisions, and behaviors relating to health [35]. This qualitative study aimed to describe family influences on PrEP use by AGYW in a PrEP demonstration project in three African cities to improve our understanding of how programmes can leverage family relationships to support PrEP use.

## Methods

### Overview of the POWER trial

The POWER (Prevention Options for Women Evaluation Research) demonstration project assessed PrEP uptake, adherence, persistence, and HIV protection when offered as part of standard of care (SOC) services [6]. It aimed to develop scalable and setting-specific PrEP delivery strategies for AGYW in Africa. Between 2017–20202 the study enrolled a total of 2550 young women from four study sites: Cape Town (CT) and Johannesburg (JHB), South Africa, and two sites in Kisumu (KSM), Kenya. Participants were eligible if they were 16–25 years old, HIV uninfected, sexually active in the previous three months, and not pregnant at the time of enrollment. The four sites offered PrEP through three delivery models: mobile outreach clinics (CT), youth clinics (JHB), and family planning clinics (both KSM sites). The study showed high PrEP uptake (93%) and modest PrEP persistence at one month (31%) and three months (20%), similar to other studies [2, 5].

### Conceptual framework

The qualitative component of the study was designed to explore different states of AGYW’s PrEP user journeys. Drawn from human-centered design, the user-journey framework highlights how participant’s needs and barriers and facilitators to PrEP use shift over time, from PrEP initiation of PrEP, to early use experiences to ongoing adherence and persistence, including pauses in PrEP use, restarts, and discontinuation [36, 37]. This framework informed the IDI sampling, development of the guides and codebook, and the analysis process.

### Qualitative recruitment

We aimed to recruit up to 75 POWER participants from each of the three cities. For in-depth interviews (IDIs), purposive sampling was used to recruit participants from six PrEP use categories, based on stages of the end-user journey and defined by clinical data and pharmacy records of PrEP refills (see Table 1) [37]. For focus group discussions (FGDs), we used convenience sampling to recruit participants who had received at least one PrEP refill during the POWER study and were attending POWER activities or clinic visits toward the end of their participation in the study [38]. All participants enrolled in the POWER trial were informed about IDIs and FGDs and indicated their willingness to participate during the informed consent process. To select the IDI participants, a list of randomly ordered cohort participants meeting the criteria for each PrEP use category was sent to site by the Qualitative Data Management Center every 2–4 weeks along with a target number of participants to enroll from each category, in order to ensure that the sample included POWER participants from throughout the enrollment period. Participants who had consented were contacted telephonically, one by one in the order of the list provided, and invited to participate, until the target number of interviews was reached. FGD participants were approached during their study visits and invited to take part in the FGDs. AGYW who had participated in the IDIs were excluded from the FGDs.

**Table 1:** Description of participant PrEP user categories.

Participant categories	Category description (Selection criteria based on clinic and pharmacy records)
Early Acceptors	Initiated PrEP at enrolment and completed the Month 1 visit
Early Refusers	Declined PrEP at enrolment OR initiated PrEP at enrolment but did not return for month 1 visit. (May or may not have started PrEP later).
Persisters	Initiated PrEP at enrolment and continued PrEP over 3 or 6 months with no gaps in pill coverage.
Non-Persisters	Initiated PrEP at enrolment and continued through Month 1, but was late for, missed, or declined PrEP refills at the Month 3 or Month 6 visit.
Restarters	Initiated PrEP, then had a break in PrEP use for more than 30 days before a PrEP refill at a later clinic visit.
Special Cases	Had circumstances or perspectives that stood out and whose experiences could inform PrEP delivery (e.g., sero-convertors)
FGD Participant	Initiated PrEP, had at least one PrEP refill, and attended clinic visits or site activities toward the end of their study participation

### Data collection

Both IDIs and FGDs used semi-structured interview topic guides based on the end-user journey framework. IDIs explored participants’ PrEP use experiences, including their awareness and interest in PrEP; the barriers and facilitators to PrEP uptake and use; the role of family, peers, intimate partners, and community influence on PrEP use; and support at relevant stages in their PrEP use journey. IDIs were held in a private room at the various study sites. Each interview took approximately 60–90 minutes [38].

FGDs comprised between 3–7 participants and explored social influences on PrEP use and PrEP experiences, including a reflection on how their experiences compared to that of the young female character in a vignette during various stages of that character’s PrEP user journey [38]. Participants also completed a social mapping exercise to reflect on the people in their circle of influence who were the most and least influential in their PrEP journey [39]. FGDs took between 45–120 minutes.

IDIs and FGDs were conducted in the local language(s) by trained female research assistants. A facilitator and a note-taker were present at each FGD. All discussions were audio-recorded, then transcribed and translated into English by trained transcribers [38].

### Data analysis

Dedoose version 6.1.18 was used to code and organize data. Four analysts coded data following a codebook developed iteratively around the end-user journey framework [36]. Each transcript was coded by one analyst. The analysis team periodically compared sections of independently coded transcripts using the Dedoose Training Center and received an average intercoder reliability agreement of 0.76 [38, 39]. Disagreements were discussed and resolved by the analysts.

**Fig 1:**
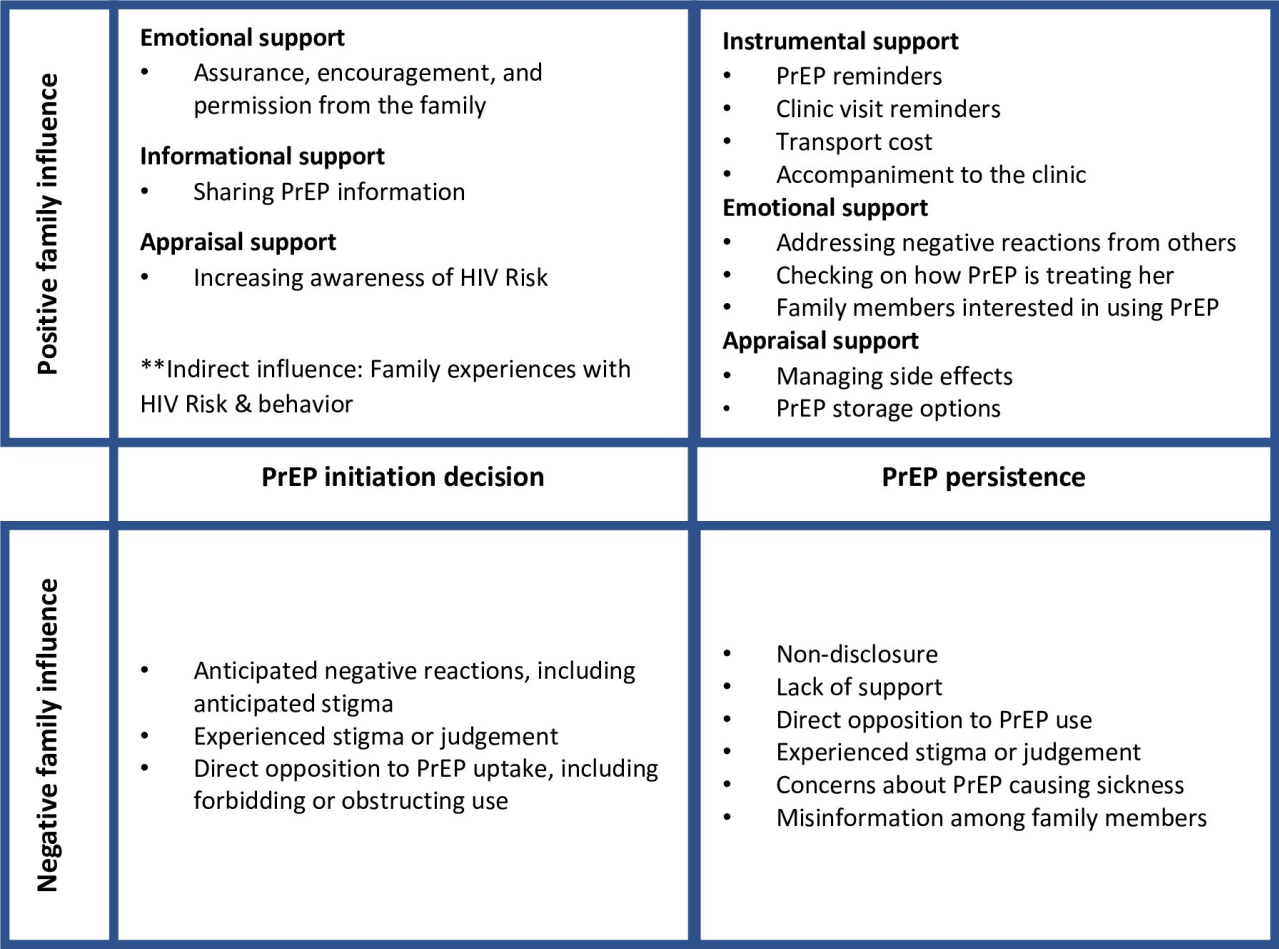
Conceptual framework: Family role in AGYW PrEP journey.

The current analysis on family influence used a thematic approach, rather than directly following the end-user journey framework, because the guides did not explicitly focus on family influence at each journey stage. We sought to examine family influence in PrEP uptake, the role of family support during PrEP use, experiences of family opposition to PrEP use, and PrEP non-disclosure to family members. Throughout the analysis, we focused on the types of family support or opposition participants experienced and how they felt it facilitated or hindered PrEP initiation and persistence. Fig 1 depicts key themes on the role of family in AGYW’s PrEP use at the two stages of the end-user journey that arose in the thematic analysis: initiation and persistence.

Specific relevant codes (e.g., family and disclosure) were selected and a team of social science researchers from South Africa and the United States completed memos for each participant and each FGD from the code reports. The team held regular meetings to discuss memos and themes emanating from the data. Memos were grouped by participant’s end-user journey category and then themes were identified within and across categories; a matrix was created to assess the patterns, similarities, and differences between sites and categories for each theme. Quotes and examples are provided for each theme using pseudonyms to conceal participant identity and ensure anonymity of the IDI and FGD participants. In the IDIs, pseudonyms were selected by the first author and applied during the analysis. In FGDs, pseudonyms were chosen by participants as preferred names to be used during the group discussions. Participant’s age, data collection category, and city are also provided to characterize their experiences.

### Ethics statement

The POWER study received ethics approval from the following institutional review boards: the University of Washington, University of the Witwatersrand, University of Cape Town, Kenya Medical Research Institute. Written informed consent was obtained for participation in the qualitative component at all sites. Kisumu and Cape Town study sites received parental consent waiver for 16- and 17-year-olds and the Johannesburg site only enrolled participants who were 18 years old and above.

## Findings

### Demographic characteristics

A total of 136 AGYW were interviewed (Table 2), including 104 IDI participants and thirty-three FGD participants; one participant participated in both activities. The majority (68.0%) of participants were between the ages of 20–25 years old. In South Africa (CT and JHB sites), almost half (48%) of the participants lived with their parents. Over 80% of participants in South Africa were not married but had sexual partners. In Kisumu, almost half (40%) of the young women were married and lived with their husbands.

**Table 2:** Demographic characteristics of the POWER qualitative participants (N = 136).

Characteristics		Kisumu (n = 50)	Cape Town (n = 42)	Johannesburg (n = 44)	Total (n = 136)*
		N (%)	N (%)	N (%)	N (%)
Age **					
	16–19	13 (26%)	23 (55%)	7 (16%)	43 (32%)
	20–25	37 (74%)	19 (45%)	37 (84%)	93 (68%)
Current relationship status					
	Single, no partner	3 (6%)	8 (19%)	5 (11%)	16 (12%)
	Single, with partner	25 (50%)	34 (81%)	37 (84%)	96 (71%)
	Married (husband has one wife)	20 (40%)	0 (0%)	2 (5%)	22 (16%)
	Widowed or Divorced/separated	2 (4%)	0 (0%)	0 (0%)	2 (1%)
Highest level of education					
	Secondary school not complete	28 (56%)	18 (43%)	9 (20%)	55 (40%)
	Secondary school complete	11 (22%)	10 (24%)	25 (57%)	46 (34%)
	Attended college or university	11 (22%)	14 (33%)	10 (23%)	35 (26%)
Currently in school					
	Yes	11 (22%)	29 (69%)	21 (48%)	61 (45%)
	No	39 (78%)	13 (31%)	23 (52%)	75 (55%)
Sources of income					
	None	33 (66%)	31 (74%)	13 (30%)	77 (57%)
	Formal employment	5 (10%)	4 (10%)	13 (30%)	22 (16%)
	Informal employment	8 (16%)	1 (2%)	6 (14%)	15 (11%)
	Social grant	0 (0%)	5 (12%)	1 (2%)	6 (4%)
	Other***	3 (6%)	1 (2%)	11 (25%)	15 (11%)
Who currently lives with					
	Parent(s)	15 (30%)	31 (74%)	19 (43%)	65 (48%)
	Other family	16 (32%)	9 (21%)	10 (23%)	35 (26%)
	Husband/sexual partner	20 (40%)	0 (0%)	5 (11%)	25 (18%)
	Friend(s)	1 (2%)	0 (0%)	5 (11%)	6 (4%)
	Alone	2 (4%)	2 (5%)	6 (14%)	10 (7%)
Data collection category					
	Early Acceptors IDI	6 (12%)	7 (17%)	12 (27%)*	25 (18%)*
	Early Refusers IDI	10 (20%)	6 (14%)	7 (16%)	23 (17%)
	Persisters IDI	7 (14%)	3 (7%)	6 (14%)	16 (12%)
	Non-Persisters IDI	8 (16%)	7 (17%)	8 (18%)	23 (17%)
	Restarters IDI	2 (4%)	3 (7%)	0 (0%)	5 (4%)
	Special Cases IDI	5 (10%)	3 (7%)	4 (9%)	12 (9%)
	Focus Group	12 (24%)	13 (31%)	8 (18%)*	33 (24%)*

*One participant at Wits RHI participated in both an early acceptors IDI and a focus group

### Family influences on the decision to initiate PrEP

#### Motivation

Participants from all study sites described how families featured in their thought process when deciding to initiate PrEP. Family experiences of risk behaviour and HIV, for example having family members living with HIV or loss of family members due to HIV-related complications, motivated most participants to initiate PrEP. Participants feared contracting HIV ‘from’ their family members or ‘like’ their family members did. Betty (age 19), an early PrEP acceptor from Kisumu, described this fear (and showed a misunderstanding of HIV transmission) in her remark: “*My aunt is [HIV] positive*, *and most of the time I take [spend] time with her*, *I felt I could be at risk because I can handle things carelessly and get infected*.” Similarly, Nondwe (16), an Early Acceptor from Cape Town, recalled: *“My cousin recently got infected with HIV*. *So*, *I heard that*, *and I didn’t want to be in the same situation*, *to be like her*. *I decided to look for help*.” Family members’ engagement in risk behaviour prompted other participants to initiate PrEP. For example, Tebogo (20), an Early Refuser from Johannesburg, eventually decided to start PrEP because she wanted to teach her younger sister about making wise decisions. For participants like Tebogo, the decision to take PrEP was about changing the status quo in their families, taking care of themselves, and serving as role models.

#### Anticipated experiences of disclosure to family and subsequent reactions

In addition to considering their family’s HIV-related experiences, participants reported ambivalence about PrEP uptake and disclosure as they thought about the circumstances under which they would take PrEP and the potential negative reactions they might receive from family members. This led to fear and stress about these reactions and uncertainty about whether they would inform family members about their decision to initiate PrEP. As participants reflected on conflicting thoughts and emotions they experienced when making this decision, they highlighted anticipated stigma from family members.

“Well, for one uh the people around me, my friends and family, they wouldn’t understand I think it would come off as if I’m already HIV positive… So that was one of my main concerns.” (Tebogo, age 20, Early Refuser, Johannesburg)

“I had stress regarding that [decision]…My grandmother will say, ahh she is lying, what she is saying to me is not true, she is sick. So, I had that thing, I don’t know how I will do it, should I take it [PrEP] or not, but I also had that thing that says, you know what, I don’t want to be like my mother [who died of HIV]… so if I need to explain at home and I will explain.” (Mbali, age 20, FGD participant, Johannesburg)

Despite these concerns about family reactions, participants mostly made an independent decision to accept PrEP, with the attitude that they were doing it for themselves and would deal with family at a later stage. This was most common among participants in the Early Acceptor category, who accepted PrEP the first time it was offered to them, but was observed among some participants in all categories.

#### Need for advice or permission from mothers

Not all participants made independent decisions to accept PrEP. Some participants had uncertainties and had questions about PrEP, and its potential implications. As a result, some, including most Early Refusers, deferred PrEP uptake because they felt the need to engage their family, especially their mothers, to seek advice, assurance, or permission to use PrEP. Noloyiso (21), an Early Refuser from Cape Town, remarked:

‘‘I felt like I had confusion and had a lot of questions that I asked myself if I should take or not? Will I be taking pills every day? What will I tell my parents about these pills? Perhaps my mother will not understand this thing; she might have a different perception about PrEP, and I must explain all these to her. So that’s why I decided to go talk to her first before making a decision.”

Many South African participants reported that their mothers’ reactions were more important than those of other family members. The reasons cited for the importance of mothers were that participants lived with them, mothers were the primary decision-makers in other aspects of the participants’ lives, and participants did not want them to be shocked or concerned by seeing them taking pills. For these participants, mothers’ opinions and attitudes towards PrEP played a critical role in whether they accepted PrEP or not. This was particularly common among young women in Johannesburg who were mainly still in school and dependent on their parents. Nomali (18), a Non-Persister from Johannesburg, stated:

“… I needed a decision from my mother before I could take them so that when I take them [PrEP pills], I don’t hide them so that I can be comfortable even if I leave them at home…Because like my mother… when she takes a decision, I accept it, even if she said I should not take them, I was not going to take them.”

#### Types of family support for PrEP initiation

Emotional support, in the form of encouragement, was the most common type of support young women received from mothers and other family members during the PrEP uptake decision, but informational and appraisal support were also provided in a few cases. Among both Early Refusers and Early Acceptors, those who engaged their families in their PrEP decision usually reported that family members encouraged them to use PrEP. Noloyiso (21) expressed her mother’s response to her PrEP disclosure:

‘‘She was very understanding. She never questioned negatively about PrEP. I explained to her and gave her a pamphlet to read. So, she encouraged me to go for it… I was relieved; I felt so relieved.” (Early Refuser, Cape Town)

Participants commonly indicated that family members initially did not have PrEP information but still agreed for young women to use it. A few family members took it upon themselves to learn more about it by asking friends, the internet, and nurses. Once they were knowledgeable, some became even more supportive. A few participants, however, reported that family members already had information about PrEP safety and efficacy. In such contexts, family members shared information on PrEP and how it works with young women who were still deciding to take PrEP, endorsed PrEP use, and used such information to educate other family members who had negative reactions. Mwiti (14), a Non-Persister from Kisumu, described how receiving informational support for PrEP from her mother helped her decide to initiate PrEP. She articulated: “*I went and asked my mom… She told me that it had been proven that it works*. *I also got encouraged by that*.*”*

In some instances, mothers actively influenced participants’ decision to start PrEP through appraisal support. Marie (18), a young Kenyan woman who initially refused PrEP, reported that she did not see the need to take PrEP because she trusted her boyfriend. Marie also stated that she did not trust that she would be able to take PrEP consistently. Marie’s mother encouraged her to initiate PrEP as she knew that Marie was dating, stating, ‘*you can never be too safe…*’ and this influenced Marie’s decision to start PrEP. Other mothers were aware of the HIV risk young women were facing every day based on their jobs, the frequency of gender-based violence, cheating sexual partners, and partners living with HIV. One participant remarked: *‘My mother was very supportive and encouraged me to go for PrEP since my husband was already HIV positive; she wanted me to protect myself from infection and also protect my unborn child*.” (Mercy, age 24, FGD participant, Kisumu). Similarly, Zawadi (22), an Early Refuser from Kisumu, stated: ‘*She [mother] knows that I am a young girl*, *and the world today is not like theirs then*, *and I also work in the field and am at risk*.*’*

#### Family opposition to PrEP initiation

A few young women also reported opposition from family members when they communicated their intention to initiate PrEP. Nwabisa disclosed to her mother before taking PrEP; however, her mother was concerned about the effect PrEP would have on her health and thought that there was no difference between ART and PrEP:

‘At first, I told her that there is a pill like this [PrEP]. Then she said that “you should come with this pill so I can see it.” Then I came back with the pills. She said, “No man, if you are going to take these pills every day now when you reach the age of 25, (imagine) how will they have affected you internally”. I then explained to her that they explained to us that we could stop taking PrEP at some point and start taking it again and start using it. I explained to her, but she is against it because she says it is the same as taking ARVs.’ (Nwabisa, age 17, Restarter, Cape Town)

Nwabisa was unhappy with her mother’s reaction but also thought, ‘*she [her mom] has her life and I have my own life’*. She continued taking PrEP regardless of her mother’s response.

### Family support during PrEP use

#### Instrumental support

Pill taking and clinic visit reminders were the most cited form of instrumental support from family members, particularly mothers. Minky (24), a PrEP Non-Persister from Cape Town, remarked: *‘she [mother] doesn’t even set the alarm*. *If it is 12 o’clock [noon]*, *she will ask if I have taken the pills*, *then I will remember or tell that I have already taken the pills*.’ Similarly, Betty (19) received PrEP reminders from her entire family: “*Mum and dad when they notice I have not taken they tell me*, *even my brothers they remind me five minutes earlier before the time of taking”* (Early Acceptor, Kisumu). For Asanda (20), a Non-Persister from Cape Town, her mother reminded her about her clinic visit, stating, *‘My mother is thoughtful… she is so fearful that I might get the diseases that we are facing currently*. *She always reminds me and tells me don’t forget to go to the clinic*.*’* Participants reported that such reminders helped them adhere to their PrEP routine.

Financial assistance was another type of instrumental support participants received from their mothers. They described instances where they had challenges getting to the clinic because they did not have transport money or time. Mothers offered transport money or to pay for PrEP refills from more convenient retail pharmacies. While financial support was well received and appreciated by most participants, one participant reported that she felt like a burden to her mom and felt "too old" to be asking for money from her parents. As a result, she missed the clinic visit because she was too embarrassed to ask for money.

Other forms of instrumental support the participants received from their families included accompaniment to the clinic, help with PrEP storage and help with communication with the clinic. Instrumental support was also a way for parents to get more PrEP or study information in order to continue supporting the young women using PrEP. Thuli (19), an Early Acceptor from Kisumu, stated that both her parents accompanied her to a clinic to collect her PrEP refills. After speaking with the nurses, her parents told her that it was okay to continue participating. Another Early Acceptor from Cape Town, Nandipha (16), aligned her PrEP clinic visit with her aunt’s ART clinic visit and they travelled together to the clinic. Family members also helped AGYW with the safe storage of the PrEP pills at home. One participant reported storing her pill bottles at her sister’s place because she had not disclosed it to others. Noxolo (20), a Non-Persister from Cape Town, did not have a mobile phone but felt supported by her sister who allowed her to use her phone to receive communication from the study staff.

#### Emotional support

Participants commonly mentioned that mothers provided encouragement for PrEP use due to their concerns about high HIV risk in the community. In many instances, mothers did not understand PrEP efficacy and safety but endorsed it if their daughters felt it was something they needed and was safe for them to use. Some of the mothers’ responses as reported by participants, are listed below:

‘… if what you are taking is going to work for you, it’s fine, but as long as I will be safe.’ (Zukiswa, age unknown, FGD participant, Cape Town)

‘…she [mother] said that I shouldn’t use it [PrEP] if at all it is something bad. She said that I can use it [PrEP] if it is something useful.’ (Zuri, age 25, Non-Persister, Kisumu)

Mothers also offered emotional support to young women who experienced negative reactions from other family members or community members and advised them on how to cope with negative PrEP related comments. Yolisa (20), a Non-Persister from Cape Town, reported that her family made negative comments about her using PrEP, mistaking it for ART. Her mother stepped in: “*She (mother) supported me and told me that I should not be ashamed wherever I am in my time of taking pills*, *I should take it*. *Irrespective of what other people say*, *I should think of the reasons why I am using PrEP*.*”* This helped her build resilience against negative reactions from others. Finally, some participants reported that when they disclosed PrEP use, some family members e.g., sisters and cousins were interested and wanted to use PrEP themselves. One participant from Kisumu spoke about her sister who initially discouraged her PrEP use, but later encouraged the participant to take PrEP. Later, her sister also initiated PrEP. For this participant, this meant that her sister understood and endorsed PrEP use, which was motivating for her. Participants also appreciated questions as simple as ‘*how is PrEP treating you*?’ as an expression of care and support.

#### Information support

Although less frequently, mothers also offered young women guidance and advice such as problem-solving on dealing with PrEP side effects and tips on PrEP storage. Nosipho, a Persister from Cape Town reported a house break-in where her pills went missing. Following the incident, her mom advised her to store her PrEP tablets at different locations in the house to ensure that she does not lose all her pills. Mothers also offered practical advice on how to deal with family members or partners opposing PrEP use:

“[My mother] said if [my partner] doesn’t accept my decision, I should not even take the tablets to show him; I should keep them inside the house for them to be safe.” (Palesa, age 20, Persister, Johannesburg)

### Family opposition to PrEP use

Although most participants reported receiving support from their families during PrEP use, a few participants reported a lack of family support and/or direct opposition to their using PrEP. Two key reasons emerged as to why families opposed PrEP use among young women. First, they doubted PrEP, confusing it with ART, and misattributed PrEP pill taking to being treated for an HIV infection. Nomvuyo age (20), an Early Acceptor from Cape Town, had a difficult time with her family at first. Her family’s disbelief that PrEP was for HIV prevention caused conflict as they thought that they recognized the pills as the same ones used for ART (since they look the same) and thought Nomvuyo was HIV positive and being dishonest about her status. Assumptions that young women were taking these pills because they were HIV positive were common in instances where young women had not disclosed about their PrEP use, and family members found out accidentally.

Second, family members feared that PrEP would make participants sick, cause side effects, or give them HIV. Wawira age (18), an Early Refuser from Kisumu, stopped taking PrEP because her father attributed her heart pain to PrEP. Similarly, Lolo (age unknown), an Early Refuser from Johannesburg, reported that her mother asked her to stop taking PrEP because she thought it would make her sick.

As mentioned above, PrEP opposition by family members caused some participants to stop PrEP completely or use PrEP covertly, as Lolo decided to do. Lolo reported taking PrEP well despite having to hide it. However, she admitted it is challenging to take PrEP with her family around. She used to set the alarm to remind her, but she stopped because her family asked about it. She then took PrEP in her room when a popular TV soap opera starts. Opposition from family did not stand in the way of her use of PrEP.

It appears that the unsupportive parents often relied on (mis-)information they received from friends and community members. When family members gained access to accurate PrEP information, they became supportive. Nomvuyo stated that as community members began to share their PrEP use, her family began to believe in this preventive strategy and showed understanding. At the time of the interview, Nomvuyo reported that her sister had gone to get PrEP the previous day.

### PrEP non-disclosure to family members

A few participants could not experience support or opposition because they did not disclose PrEP use to their families. Some of these participants explained that they did not disclose because they did not reside with their family members. Anna (23), an Early Acceptor living in Johannesburg, had not disclosed PrEP use to her parents living in Zimbabwe as she did not want to disclose over the phone. She planned to do it in person next time she visited her parents.

Anticipated stigma did not only influence PrEP uptake decisions and PrEP use, as shown in earlier sections, it also contributed to non-disclosure of PrEP use. Some participants chose not to disclose PrEP use because they did not want to reveal that they were sexually active or engaging in risky behaviour. Annette had not disclosed to any of her family members because:

“Some people would not understand why I am taking PrEP. They would think I am taking it because I am HIV positive… My parents are very strict, they would think things like I am having sex, ja, so it’s gonna be hard for them to understand why I am taking PrEP.”

Aviwe (22), an Early Acceptor from Cape Town, did not reveal her PrEP use to anyone. She said she did not want her family assuming that she was taking ART. Furthermore, she thought it would be insensitive of her to talk about HIV prevention to her brother, who was already living with HIV, and doubted that he would support her. Aviwe also expressed the difficulties in telling other people about PrEP; she would not know what to say. This suggest that some participants may need guidance on how to tell their families about PrEP.

Non-disclosure typically led to PrEP use challenges similar to those arising from family opposition, causing participants to miss doses in settings with no privacy. For example, Aviwe had difficulties taking PrEP at home; as a result, she took PrEP at work. Nomali had disclosed PrEP use to her supportive mother but reported that she missed her PrEP doses when she attended a family funeral because she had not told her aunt. Another participant spoke about the challenges she faced taking PrEP when her sister was around. As mentioned earlier, some young women had not disclosed PrEP, but family members found out accidentally about their PrEP use; this led to the assumption that young women taking PrEP were HIV positive.

## Discussion

In this qualitative study, we explored family influence on PrEP initiation and persistence among AGYW in a PrEP demonstration project in three African cities. Our findings show that family members, particularly mothers, play a crucial, direct and indirect, role in PrEP uptake decisions and can support PrEP use. Three main findings emerged from this study: 1) PrEP decision-making is influenced by motivation to prevent HIV based on family experiences of HIV but offset by fear and stress caused by anticipated stigma from family members; 2) Disclosure to family members, especially mothers, and subsequent support are important facilitators of PrEP initiation and persistence for AGYW; 3) For a smaller number of participants, receipt of support is impeded by family members’ limited information about PrEP safety and efficacy. In most instances, access to trusted information resulted in family support even if there was initial resistance.

PrEP uptake decisions among study participants were characterised by mixed emotions. They were motivated to use PrEP by personal experiences of family members dying or living with HIV. Participants’ PrEP acceptance seemed to sometimes be motivated by wanting to avoid becoming like other family members who were directly affected by HIV. However, they also experienced fear and stress related to how their family would respond and anticipated HIV stigma from family members. The fact that PrEP disclosure also required indirectly disclosing that they were sexually active, which is a taboo for AGYW in most African societies. Decision making based on family’s lived experiences of HIV and anticipated HIV-related stigma from family members may distort AGYW’s own judgment of whether or not they decide to accept PrEP and make it challenging to assess their HIV risk. Poor assessment of HIV risk may lead to ineffective use of PrEP [24], if a young women’s motivation to take PrEP is not aligned with their epidemiological risk [24]. However, since young people tend to have an ’optimism bias’ and lack of saliency [2], having the experience of family members with HIV could help them recognize the reality of HIV and its consequences, leading to them to take action to protect themselves. In contrast, anticipated stigma from family members could lead AGYW to delay PrEP initiation or delay PrEP disclosure. Non-disclosure of PrEP use can result in challenges with PrEP storage and adherence [11].

Disclosure to family members, especially to mothers, was important for most AGYW, who often felt the need for their mothers to be aware of and approve of their PrEP use. This was true across sites, despite the fact that Kenyan participants were more likely to live with their male partners, and less likely to live with parents, than those in South Africa. These findings are consistent with those from a study conducted by Scorgie et al. [2021], where AGYW disclosed to female family members (mothers and sisters) to receive support for their PrEP use [31]. Our findings highlight that mothers are important sources of instrumental, emotional, informational, and appraisal support during PrEP uptake decision-making and PrEP use. Before PrEP initiation, mothers generally advised and endorsed PrEP uptake and helped AGYW recognize their vulnerability to HIV. During PrEP use, they offered reminders, transport money, advice, encouragement, and information. Our findings are similar to those on the significant role families play in antiretroviral therapy [19]. Young women valued the validation received from their mothers the most, citing that it assured them that they had made a good decision, with some AGYW reporting that it facilitated their PrEP adherence. Instrumental and informational support from family members also helped AGYW address specific challenges they faced. This suggests the potential benefit of programmatic or counselling support to help them identify the types of support they require and then strategically disclose to family members who they believe will provide those types of support.

Although it was less common, some family members did not have sufficient information about PrEP safety and efficacy. Family members with limited or no information about PrEP either supported AGYW based on the information given by AGYW, or they opposed PrEP use due to safety concerns. This opposition acted as a substantial barrier to providing support to the AGYW in their lives. Prior PrEP awareness and information among family members enabled participants to get informational support from family in addition to emotional support, which helped them make decisions about uptake. These findings are consistent with HIV treatment studies showing that a lack of knowledge and understanding of the therapy resulted in social stigma and discrimination, and that access to information resulted in better support [40]. In this study, family members who made an effort to find out more about PrEP often became more supportive once better informed. Thus, raising PrEP awareness and providing information to adults in communities more generally could enhance PrEP uptake, initiation and persistence while minimizing PrEP opposition.

Overall, we found that PrEP programs would benefit from recognizing family members, most especially mothers, as an important resource supporting PrEP uptake and use among AGYW. While involving mothers in PrEP decisions could reduce stigma and foster social support and potentially lead to better sexual risk outcome [41], previous research highlights potential barriers to parental involvement and support for PrEP use among AGYW. One, as indicated earlier, PrEP use indirectly discloses engagement in sexual activity, and sex in adolescents is frowned upon. Discussions about sex and parental support for PrEP use could be interpreted as an implicit approval for unprotected sex among AGYW [42]. Consequently parents may avoid such discussions without recognising that doing so increases their daughter’s HIV risk [42]. Two, PrEP was initially targeted towards high-risk populations such as sex workers, rather than a tool for anyone who wants to protect themselves from HIV. The resulting stigma associated with PrEP use could be a key challenge to parental support.

Results from this research, along with prior literature, have several implications for AGYW PrEP programming. Firstly, the study underlines that PrEP programming targeting AGYW should engage family members as critical stakeholders. Engaging parents is particularly important in the context of ongoing ethical and legal debates, in South Africa and throughout the region, about children’s legal rights to independently access sexual and reproductive health (SRH) services such as PrEP. Failure to engage parents and ensure acceptability risks resistance to PrEP use, reduced access through refusal to consent, and hostility to health workers who provide PrEP [43]. Since mothers clearly had the strongest influence on participants’ PrEP use in this analysis and another social map analysis conducted as part of the POWER study [39], mother-daughter interventions could be offered to bolster PrEP support. An example is the IMARA intervention, currently being tested in Cape Town [44]. PrEP programmes could also provide factual information about PrEP and its benefits through community and family-level education sessions and materials to assuage common concerns about PrEP safety and clarify the difference between ART and PrEP, thereby reducing PrEP stigma and increasing the chances that PrEP disclosure is received positively.

The study suggests that PrEP should be promoted as a tool for empowerment in preventing HIV rather than for populations at high risk of HIV. Efforts should be made to address sociocultural barriers that prevent discussions on sexuality and HIV [45]. We also recommend educating mothers on these topics to safeguard their daughters’ sexual health. PrEP programmes could also strengthen family support for PrEP by creating awareness of family members’ roles in PrEP uptake decision-making, discussing common barriers to AGYW’s PrEP use, and discussing ways for family to help overcome those barriers and facilitate PrEP adherence and persistence. AGYW have suggested that the PrEP information to their social networks should be provided by healthcare providers to remove the burden on themselves [39].

Secondly, the important role of maternal support suggests that providers should help AGYW think about what support they need to take PrEP and whether disclosing to their mothers could help them gain that support. However, some participants expressed difficulties associated with disclosure and how to communicate PrEP information to their families, with some who stopped PrEP when faced with negative reactions. Therefore, programmes should equip AGYW themselves with information and tools to facilitate PrEP discussion with strategically chosen family members and help with PrEP disclosure should they choose to do so, including disclosure tactics and coping mechanisms if they face negative reactions.

Thirdly, to address AGYW’s fear and anxiety associated with PrEP decision-making, PrEP initiation counselling should screen for and address family-related concerns about PrEP use. This will allow counsellors to identify anticipated stigma and equip AGYW with information, materials, and skills to deal with it. Velloza et al. [2020], also support the need of clinic-based discussions as strategy to address anticipated and experienced stigma [11].

A limitation of this study is that we did not include the perspectives of family members in the qualitative assessment. Participants who agreed to join the qualitative component may have had more positive experiences than those who did not agree to participate. AGYW who had the greatest challenges with PrEP likely did not return to the clinic at all and thus could not be reached for IDIs. Due to a mixed PrEP-use patterns among FGD participants there is a possibility that participants responses were colored by social desirability bias, we took steps to prevent this by using interviewers who were not engaged in the PrEP service delivery under the POWER study and, in FGDs, by using character vignettes to allow participants to share experiences and opinions without claiming them as their own.

## Conclusion

This study adds to the literature by describing the types of support that families provide during PrEP initiation and persistence and how AGYW perception of such support can promote PrEP use. Our findings show that families, particularly mothers, can play an instrumental role in facilitating PrEP use. PrEP programmes should leverage family support by providing basic PrEP information about its safety, efficacy, and benefits to families. This way family members can be informed and confident about supporting AGYW to use PrEP successfully for HIV prevention. To support AGYW to start and continue to take PrEP, programmes should also provide additional counselling to AGYW on whether and how to disclose, and on strategies to address anticipated stigma and negative reactions from family members.
